# Principles of Researching Health Disparities in Longitudinal Cohort Studies Enrolling Children

**DOI:** 10.3389/fped.2021.627298

**Published:** 2021-11-19

**Authors:** Carl V. Hill, Steven Hirschfeld, Nathaniel S. Stinson

**Affiliations:** ^1^Alzheimer's Association, Chicago, IL, United States; ^2^Uniformed Services University of the Health Sciences, Bethesda, MD, United States; ^3^Division of Scientific Programs, National Institute on Minority Health and Health Disparities, Bethesda, MD, United States

**Keywords:** disparities health, longitudinal studies, child health, epidemiology, health justice

## Abstract

Health disparities are defined on the basis of specific populations that, when compared to the general population, have a significant disparity on the rate of disease incidence, prevalence, morbidity, mortality, or survival. People that experience health disparities can be defined by multiple criteria. As the diversity of the United States broadens and increases, research on the origins and causes of health disparities becomes more important to support a healthy general population. Children are particularly sensitive to and vulnerable to health disparities due to the potentially life long consequences of events during periods of critical organ, intellectual and social development. The concept of health justice whereby each individual has the opportunity to realize their full health potential can only be realized with proper understanding and relevant data to frame practice, policy and actions. The National Children's Study (NCS) was a longitudinal birth cohort study designed to incorporate the principles of the Federal Collaboration on Health Disparities Research in consultation with subject matter experts, community representatives, and ongoing evaluation to ensure high quality and relevant data on factors that impact health outcomes. The NCS developed and tested a model of enrolling a diverse population, capturing and integrating data using a life course framework, constructing individual profiles, then aggregating individuals into groups based on profiles and outcomes. This approach is applicable to other longitudinal cohort studies.

## Introduction

The purpose of this document is to describe a framework for data capture in longitudinal research studies addressing health disparities in children.

The study of the susceptibility and health effects of environmental and social factors on vulnerable populations is not new or even recent. Environmental exposures as a cause of illness for an identifiable group was described by Sir Percival Pott in 1775 in his description of scrotal cancer among English chimney sweeps (1).

The typhus outbreak in Silesia described by Rudolf Virchow in 1848 was not limited to identification of the immediate microbiological cause but placed the epidemic in the context of economic, social, and cultural factors that affected the Polish minority that were primarily affected. Virchow recommended both short term remedies such as contact tracing and isolation and long term changes to prevent further outbreaks not only of typhus but also dysentery, measles, and pulmonary tuberculosis through major political and social reform. He argued that the endemic medical conditions were “artificial” and preventable. After filing his report, Virchow and a colleague started a journal called *Medical Reform* and promoted the concept of Social Medicine with the premise that the health of the population should be a matter of utmost importance to the state and that social and economic conditions have a “decisive effect” on health and disease. He further proposed that those effects should be subjected to scientific investigation using statistical analysis as a measurement standard (2).

As understanding and methods evolve, new opportunities to prevent what Virchow termed “artificial” conditions are the topics of ongoing research, particularly through longitudinal studies. Examples include the effects of birth weight, nursery care, family mental health, and social class on adult outcomes (3–10). The conventional paradigm is to analyze the data and document correlations between characteristics, exposures, and outcomes, although identification of combinations of factors and use of specific statistical methods can result in better curve fitting (4). Furthermore, the precision and validity of data analyzed retrospectively compared to prospective data collection varies but generally favors prospective data collection for greater precision and quality (11).

## Defining Health Disparities

The United States Congress defined “health disparities” in the Minority Health and Health Disparities Research and Education Act of 2000 (Public Law 106-525) as a particular population that experiences a health disparity, “if, as determined by the Director of the Center (National Institute on Minority Health and Health Disparities) after consultation with the Director of the Agency for Healthcare Research and Quality, there is a significant disparity in the overall rate of disease incidence, prevalence, morbidity, mortality, or survival rates in the population as compared to the health status of the general population.”

Thus there are three distinct components to the formal legal definition:

- identification of a population that has a difference in health status or a health outcome compared to the general population
- a determination of the significance of the impact by a designated official at the National Institutes of Health following
- a consultation with a designated official at the Agency for Healthcare Research and Quality

Implied in the definition is that an identified population can be defined by objective criteria and that the impact of the disease based on incidence, prevalence, morbidity, mortality, or survival rates can be measured in both the identified population as well as the general population with sufficient precision that any differences can be analyzed for significance.

To guide the development of data acquisition that can serve as informative markers for health disparities, the Centers for Disease Control and Prevention (CDC) provides its own definition of health disparities as “preventable differences in the burden of disease, injury, violence, or opportunities to achieve optimal health that are experienced by socially disadvantaged populations (12).”

The CDC definition adds two more characteristics to the concept of health disparity. One is that the population of interest is socially disadvantaged and the second is that the differences are preventable. In addition the term “significant” is no longer included but the scope of what can be measured is expanded to include the burden of disease, injury, violence, or opportunities to achieve optimal health.

Consequently, a composite legal definition of health disparity based on US Federal criteria includes:

- definition of a population that is recognized as socially disadvantaged or else establish criteria for defining a population that can be endorsed by a legally designated federal official
- selection of health outcomes based on burden of disease as measured by incidence, prevalence, morbidity, mortality, or survival rates or injury or violence or opportunities to achieve optimal health
- assessment of the impact of the selection health outcomes on the defined population and a reference population most commonly through a rate comparison
- determination if there is a difference in the health outcomes based between the defined population and the reference population that is significant, preventable, or both

The paradigm of health outcome differences between groups is used in other initiatives. For example,Healthy People 2020 defined disparity as “a particular type of health difference that is closely linked with social, economic, and/or environmental disadvantage.” (Department of Health and Human Service, 2000). Some, including the World Health Organization, have proposed measuring health inequality using “…gradients in which health is worse the more disadvantaged the population group is relative to the more advantaged” (13, 14).

Noteworthy in the US Federal definition of disparity is the dynamic quality of the process. Evidence is presented to qualified designated officials to make a determination. In this dynamic framework, the National Children's Study proposal was to take an agnostic approach to identification of potentially vulnerable populations that experience a health disparity with the perspective that all children are vulnerable to different degrees and potentially socially disadvantaged depending upon age, location, and context.

### The United States Is Increasingly Diverse

Over the past few decades, there has been a notable increase in the diversity in the US population, as summarized in Table 1 with data from the US Census Bureau. The Diversity Index, meaning the likelihood that two persons chosen at random from the same area, belong to different race or ethnic groups as defined by the Office of Management and Budget and the Census Bureau, increased in the last decade from 54.9% to 61.1%. The NCS planned its approach using the 2010 Census data with the preliminary data from the 2020 Census included in Table 1 to provide additional context and validation.

**Table 1:** Diversity in the United States.

**Population**	**Proportion 2000**	**Proportion 2010**	**Proportion 2020**
White Non-Hispanic	75.1	63.7	57.8
Hispanic or Latino	12.5	16.3	18.7
African-American	12.3	12.6	12.1
Asian	3.6	4.8	7.2
American Indian or Alaskan Native	0.9	0.9	2.9
Native Hawaiian or Pacific Islander	0.1	0.2	0.4
Age <18	25.7	24.0	22.1
Diversity Index		54.9	61.1

*Increasing diversity of the total population of the United States documenting the shifts in populations based on race, ethnicity, and age with an increasing diversity index. The diversity index is a calculated hypothetical probability of the likelihood that two persons chosen at random from the same area belong to different race or ethnic groups as defined by the Census Bureau*.

In addition to racial or ethnic minorities, the Williams Institute estimated that the number of adults who identify as lesbian, gay, or bisexual (LGB) is 4.5% of the US population, whereas an estimated 8.2% adults have reportedly engaged in same-sex sexual behavior and nearly 11% acknowledged some same-sex attraction. Transgender adults are estimated to represent approximately 0.4 % of the population (15). People with disabilities number approximately 28 %, of the population, with at least half reporting having a severe disability (16, 17).

### Building on Current Concepts of Health Disparities

In the United States, health disparities are found between those of different racial, ethnic, geographic and socioeconomic groups. For example, infants born to black women are 1.5-3.0 times more likely to die compared to those born to women of other races. Black men and women are more likely to die of heart disease or stroke than their white counterparts. People in the United States with the highest income inequality report having the fewest number of environmentally healthy days (18).

Disparities can also occur within a group that, as a whole, has poorer health outcomes. While blacks as a group have lower life expectancy than whites, it is still stratified by education level (19, 20). Additionally, though the percentage of low birth weight babies is higher for black women overall, black women living in the United States but born in Africa or the Caribbean have low birth weight baby rates similar to white women (21). Those in lower income strata, regardless of race, are exposed to different environmental hazards and social stressors than more advantaged population groups, which in turn have consequences for health (22, 23).

Additional insight is needed to determine how health disparities arise, are sustained over the life course and trans-generationally, and what factors exacerbate or protect against poorer health outcomes (24) physiological systems responding to environmental stressors, offers a framework for understanding the interplay of factors that may lead to health disparities (25, 26).

The concept of allostatic load, the wear and tear on the body caused by dysregulated behavioral or Allostatic load may increase as a result of cumulative stresses over time or due to adversities experienced during sensitive developmental periods (27–29) Differences in frequency, absolute number, or the timing in which populations experience stressors may contribute to health disparities. Stress can sometimes be at least partially buffered by biological differences, social support networks, and collective responses regarding the nature of stress (30).

Adjusting to stressors requires energy and children require more energy than adults. A newborn has approximately the same energy requirements as an adult, but the fat-free mass adjusted expenditure rapidly increases over the course of the first year to approximately 50% higher than adults. From 1 to 20 years it slowly decreases to adult values, and then remains stable for around four decades before beginning a slow decline starting around age 60 (31). Thus to maintain adequate growth and development for an individual, the supporting environment must provide sufficient energy and appropriate nutrients to address all needs including stressors that increase allostatic load. A research study interested in both growth and development and disparities must capture this information in a systematic and analyzable format.

Health conditions place stress not only on the individual, but for those in an individual's social network. For example, high rates of childhood asthma mean adult caretakers may need to take time off work, experience sleep disruptions, divert family resources into medical care, and experience additional sources of anxiety. Similarly, when adults have health problems, their ability to effectively parent becomes constrained by their own physical, mental, and psychological limitations.

Health disparities research has accelerated since a 2003 Institute of Medicine publication raised awareness about racial, ethnic, and other disparities within US health care (32) In the last two decades, heightened Federal government efforts have mirrored academic and public health interest in advancing state-of-the-art health disparities research, as demonstrated by the establishment of the National Institute of Minority Health and Health Disparities (NIMHD) and the Federal Collaboration on Health Disparities Research (FCHDR) (33).The FCHDR has five goals:

1. Identify health disparities challenges including the scientific and practical evidence most relevant to underpinning future policy and action;
2. Increase and maintain awareness about federal government efforts and opportunities to address health disparities;
3. Determine how evidence can be translated into practice to address health disparities and promote innovation;
4. Advise on possible objectives and measures for future research, building on the successes and experiences of health disparities experts; and
5. Publish reports that will contribute to the development of the FCHDR strategic vision and plan.

### Health Disparities Research Focusing on Children

The majority of health disparities research has focused on adults, and the paucity of attention devoted to children is a significant concern given the reciprocal impact of child and adult health disparities across the life course (34). Disparities occur from an interplay of multiple factors at a given life course stage and over time. Major life events such as changes in family or social situations or changes in health status of an individual or of a family member present challenges and opportunities that can alter a child's developmental trajectory (35, 36).

In health disparities research focused on children, several characteristics differ from studies focused on adults.

(1) Demographic patterns vary with disproportionately higher rates of poverty of ~21 % in children vs. ~9 % in adults, and higher racial and ethnic diversity as a result of increasing frequency of multi-ethnic and multi-racial relationships and marriages,
(2) The rapid pace of developmental changes and need for a supportive environment must be assessed in children. Adult health assessments tend to focus on health maintenance and recovery, while children are passing through windows of vulnerability and opportunity and progressively learning independence and realizing potential.
(3) Children have greater dependency on others than most adults. Parents, family, child care providers, teachers and the larger community are all influences and contributors to a child's health. Thus the health of the family and community and the health of the relationships among them and between them and the child also become determinants of children's health outcomes.
(4) The spectrum and epidemiology of health-related conditions vary with age and developmental stage. The incidence, prevalence, morbidity and mortality of phenotypes must be accounted for based on relevant factors.

### Relevance of the Concept of Health Justice

The principle of justice as applied to research is one of the three primary ethical principles established by the Belmont Report holding that research supported by the public must equitably distribute burden of participation as well as advantages of discovery (28, 37). Thus, a realization of health justice is a fundamental translational component of research that has as a goal the identification of health disparities among identifiable groups.

The principle of justice applies not only to research but to health care delivery and opportunity. Health justice is a driving force behind US Public Health policy (24).

An implied component of justice is the application of a remedy for circumstances that violate the principles and rules of what is just. Health justice, as intended in this context, is a concept that promotes all people having the opportunity to realize their full health potential, consistent with the CDC definition of health disparity. Longitudinal study data can contribute to the implementation of health justice by providing a unique look at the multiple factors that negatively impact and undermine health justices as well as factors that support and contribute to health justice and resilience. Accurate data collection can best occur through the implementation of a broadly inclusive recruitment strategy and data content development processes.

### A Comment on the Concept of Race

In the aftermath and reconstruction following World War II, the United Nations Educational Scientific and Cultural Organization proclaimed in 1950 that all people are of the same species. Since then other organizations have used accumulating evidence to declare that on a genetic basis there are no characteristics that can be used to define race using the absence of pathognomonic genetic markers and the data that the range of genetic differences among individuals within any group are greater than genetic differences between groups to establish the perspective (22, 38–49). Despite the scientific evidence, the use of the concept of race has been used for hundreds of years to discriminate among groups of people. This discrimination has severe consequences for differential environmental exposures that link to sociocultural and behavioral responses that influence population health disparities. The US Census Bureau institutionalized and continues to refine the classification of people based on race and ethnicity, a practice that has been criticized for its inherent imprecision (50). Nonetheless, data are collected and analyzed to document the dynamic and evolving diversity of the country.

## A Proposed Model to Study Disparity

**Figure 1:**
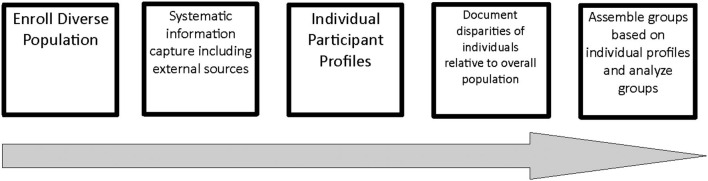
Model for documenting health disparities based on enrolling for diversity. The work flow to enroll into a study a diverse population so that sufficient numbers of potentially vulnerable individuals are included. From a diverse study population, the opportunity to capture information, identify individuals, document disparities, and assemble and analyze groups follows.

The operational key to capture groups or populations that may experience a health disparity based on the accepted definitions is to enroll into a study diverse population so that potentially vulnerable individuals are included. From a diverse study population, the opportunity to capture information, identify individuals, document disparities, and assemble and analyze groups follows. Specifically, enrollment strategies must be capable of capturing a sufficiently diverse population. Subsequently systematic data capture including integrating data from external and administrative sources such as environmental, threats to person or property, recreational, educational, access to resources, and other geographically defined information is used to construct a profile for each study participant. Health outcomes including but not limited to disease, growth, development, injury, access to professional care are then compared to the study norms and available reference data. As individuals are documented to experience a disparity, groups of individuals that experience any disparity of interest are assembled from the profiles and analyzed as a group. From these analyses, inferences, conclusions and recommendations can be generated. A schema is shown in Figure 1.

## Applying Principles to Planning and Implementation: The National Children'S Study Case Study

As an example of how to approach health disparities research in children, we will refer to The Children's Health Act of 2000. The law authorized a longitudinal study to gather “data on environmental influences and outcomes on diverse populations of children,” and to consider “health disparities among children.”

The proposed longitudinal study, known as the National Children's Study (NCS) was a longitudinal birth cohort study that was planned to adhere to federal law when defining populations experiencing health disparities, including the Minority Health and Health Disparities Research and Education Act of 2000 (Public Law 106-525) (51).

Thus, a main goal of the NCS was to identify and gather data on populations experiencing health disparities and ensure that data collection methods and materials provide comparable data for all populations under study beginning with pregnant women and ending with 21 year old adults.

The NCS was to promote health disparities research in children by providing critical research infrastructure to the field and key data on the early effects of disparities on health outcomes.

The NCS aligned with the FCHDR goals and strategies by identifying factors that contribute to health disparities across the life course. The NCS partnered with NIMHD to identify future research objectives and develop necessary measures, promote awareness of Federal government efforts, and support the conduct and publication of research studies. The NCS also addressed health disparities research recommendations put forth by the NIH's 2008 “Science of Eliminating Health Disparities Summit,” including improvement of data collection and measurement models and linking biological and non-biological determinants of health (52).

### NCS Content Development- General Approach

The Study was legally required to:

(1) incorporate behavioral, emotional, educational, and contextual consequences to enable a complete assessment of the physical, chemical, biological, and psychosocial environmental influences on children's well-being;
(2) Gather data on environmental influences and outcomes on diverse populations of children, which may include the consideration of prenatal exposures; and
(3) Consider health disparities among children, which may include the consideration of prenatal exposures.

The NCS operational goal was to capture a spectrum of outcomes that include health, disease, injury, violence, and to identify opportunities to achieve optimal health such as access to structured medical services along with home, neighborhood, school, environmental, recreational, and other factors. Subsequently, all the outcomes can be quantified and compared to the overall NCS population for any individual study participant as well as any group of study participants based on a range of criteria. Combined with genetic data, biological samples, and environmental samples, this approach provides flexibility to cluster individuals and identify populations that may experience a disparity. In addition to the internal comparison, the same comparisons can and were to be made to any validated external data.

### Utilization of a Life Course Framework

**Figure 2:**
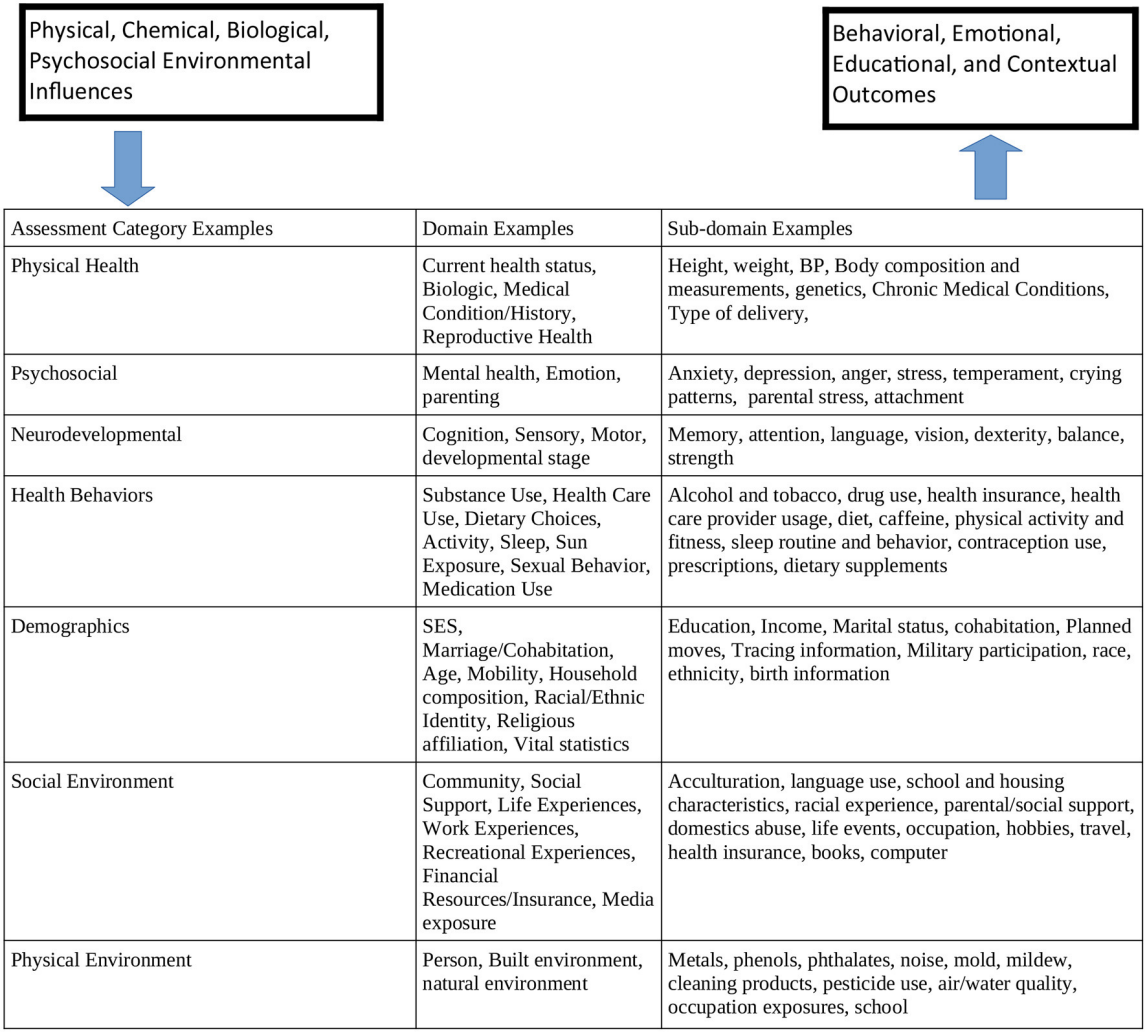
Relationship of NCS scientific goals to measurement domains. The scientific goals are summarized in the box on the upper right and the mandated outcomes are on the box on the upper left. In the middle table are examples of assessment domains with examples of sub domains that map to specific instruments.

The NCS adopted a framework based on a Life Course Perspective (53) that maps the goals to measurement domains as shown in Figure 2 (53).

The NCS plan was to collect data on markers of health disparities across all stages of child development. The life course framework supports the understanding of children's health and development, and could reveal how disparities may develop at many points along the lifecycle continuum (9, 10, 20, 34, 54–61) The longitudinal data would provide a set of measures that define the prior status of a child, an important precursor to understanding the effect of subsequent influences on later health status. By collecting data on multiple levels of influence at various times in a child's life-course, as well as measures of their health and developmental milestones, longitudinal studies like the NCS would have data that provide a timeline of the antecedents and determinants of health disparities. Some major types of developmental mechanisms for children's health captured through these data collection processes include biological programming during critical and sensitive periods, cumulative or additive effects of risks, and protective factors and developmental trajectories. This approach could thus provide a useful research paradigm in studying how disparities arise and are exacerbated or mitigated over time (62).

### Enrolling a Diverse Population

The NCS approach was to use self-reported information from the mother of a child as a marker for enrolling a diverse population and not necessarily as a precise classification of an individual child with the recognition that people have complex ancestry. The data are also of utility to maintain awareness and analyze the outcomes and effects of how a child experiences the world based on how others perceive the child and what the consequences of those experiences are.

Given the criticality of enrolling a diverse population, the NCS did what no other longitudinal did previously- establish a program for direct comparison of recruitment techniques. Beginning with selected area samples, the comparison was based on initial contact method with pregnant women or women who were planning to become pregnant. The three major approaches were door to door canvassing at dwelling units by field workers termed Enhanced Household Recruitment. In some areas the door to door canvassing was augmented by local advertising. A second approach was local advertising using a multimodal approach including billboards, print, and broadcast media termed Direct Outreach. The Direct Outreach messaging contained contact information that potential study participants would need to actively contact. A third approach was to contact local obstetrical and women's health care providers and request referrals to the NCS through the health care provider. This was termed Provider Based Recruitment. A variant on the Provider Based Recruitment was termed Provide Based Sampling and consisted of referrals through volunteer clinics plus recruitment at local hospitals and birthing centers with the addition of NCS contract staff on site to facilitate the enrollment. To reframe the approaches, one was based on initial contact at home with an unknown person, one was based on active response anywhere in a geographic area to a media outreach campaign, and one was based on referral through a trusted individual or institution in a health care context.

The results are summarized in Tables 2, 3. In brief, household or dwelling recruitment was resource intensive and provided demographics generally aligned with reference data from the geographic area. Direct Outreach captured women who were generally older, married, and more well educated than the reference population and had the lowest proportion of racial and ethnic minorities and women who did not complete high school. Using health care providers and clinics proved to be the most resource efficient and resulted in better representation of a diverse population based on demographic criteria, particularly younger and less educated women. A follow up study on comparing techniques for retention and efficient data collection was initiated but never completed.

**Table 2:** Demographics of NCS vanguard study.

	**Enhanced Household**	**Direct Outreach**	**Provider Based Recruitment**	**Provider Based Sampling**	**Combined**
Under 25	0.76	0.67	0.92	1.09	0.82
25 to 34	1.06	1.15	1.04	1.02	1.08
35 and over	1.36	1.13	1.00	0.80	1.12
Less than high school	0.72	0.29	0.63	1.04	0.60
High school / Some college	0.96	0.79	1.02	1.17	0.95
College graduate or more	1.38	1.63	1.63	0.69	1.48
Married	1.04	1.31	0.91	0.68	1.07
Unmarried	0.95	0.51	1.11	1.41	0.91
Hispanic	0.88	0.44	0.64	1.91	0.84
Non-Hispanic White	1.12	1.50	1.12	0.56	1.12
Non-Hispanic Black	0.85	0.52	1.00	1.06	0.76
Non-Hispanic Asian/Pacific Islanders	0.67	0.57	0.40	0.60	0.65
Non-Hispanic Other	1.80	1.00	4.00	4.00	3.13

*Vanguard Study Recruitment- Each column represents a recruitment technique in a defined geographic area. The value in each row represents the ratio of women recruited who gave birth and enrolled a child in the study to the US Census Bureau reference data for that geographic area. For example, the first row is maternal age at birth and the value represents the proportion of mothers in the NCS at that age as a fraction of the potential proportion of mothers living in the selected geographic area at that age. The last column is the combined values across all cohorts. The recruitment techniques are Enhanced Household = Door to door canvassing combined with local advertising. Direct Outreach = Local advertising with contact information, Provider Based Recruitment = referrals to the NCS through volunteer local obstetrical clinics, Provide Based Sampling = referrals through volunteer clinics plus recruitment at local hospitals and birthing centers*.

**Table 3:** NCS vanguard study gestational age at recruitment.

	**Enhanced Household**	**Direct Outreach**	**Provider Based Recruitment**	**Provider Based Sampling**	**Combined**
1^st^ Trimester	18%	21%	20%	54%	23%
2^nd^ Trimester	41%	38%	40%	39%	40%
3^rd^ Trimester	41%	41%	40%	7%	37%

*Comparison of pregnancy status at recruitment for different techniques. The last column is the combined values across all cohorts. The recruitment techniques are Enhanced Household = Door to door canvassing combined with local advertising. Direct Outreach = Local advertising with contact information, Provider Based Recruitment = referrals to the NCS through volunteer local obstetrical clinics, Provide Based Sampling = referrals through volunteer clinics plus recruitment at local hospitals and birthing centers*.

The experience resulted in development of a proposal for the scaled up NCS Main Study that was never implemented but was supported by recommendations found in recent publications on the topic (63, 64).

## Summary and Implications

Longitudinal studies like the NCS can address health disparities in the following ways:

- Define populations that experience disparities in disease incidence, prevalence, morbidity, mortality or survival
- Develop a research agenda and framework to identify the health determinants and unique attributes of children and their environments that influences health and health disparities
- Specifically assess and capture the CDC health disparities criteria of burden of disease, injury, violence, or opportunities to achieve optimal health for each individual and identified population
- Provide a mechanism to recognize demographic dynamics and reassess, at 5-year intervals, the most effective approaches to best identify and evaluate the populations at risk for experiencing health disparities
- Map scientific goals onto a multi-dimensional, life course developmental model of health by engaging a diverse group of experts in health disparities, other scholars, and community representatives to participate in content development, from concept to questionnaire items to other assessments that are culturally and contextually aware, to enable comparable data collection across the different subpopulations of children, including particularly vulnerable groups
- Contribute to the understanding of opportunities for health justice and maximum health potential, by providing a unique look at the multiple factors that cause, contribute to, and protect against health disparities
- Contribute to improving awareness of the diversity of people and health outcomes in the United States and serve as a resource for scholarship and training through content selection, design, analysis, and dissemination
- Document markers of health disparities through all stages of child development
- Link environmental and social exposures to health behaviors and phenotype and the presence of health disparities
- Develop timelines for antecedents to health disparities
- Identify triggering and mitigating factors for health disparities within and across populations
- Identify malleable points along the life course for intervention to reduce health disparities

By ensuring that individuals from a broad range of populations are represented fairly among study participants, data findings about the role of environmental factors on health should be relevant and applicable to populations vulnerable to experiencing health disparities. Longitudinal studies similar to the NCS would closely monitor the recruitment processes using predefined operational paradata elements to conduct close to real-time assessment of the demographics of the sampled and enrolled participants. Such assessments will allow the study to determine whether various demographic targets as markers for diversity are met. Study teams could adjust activities and resources to meet enrollment goals, a practice utilized by other survey organizations to improve the precision of survey outcomes (65).

A longitudinal study designed like the NCS will generate the information necessary to make possible policy decisions that improve the health of everyone by characterizing the complete individual within their environment, through time. This will be achieved by creating a health profile of each participant that consists of the health phenotype, the observable characteristics of the whole person at the time of assessment, plus the environmental characteristics. This framework enables data acquisition on the interactions of environment, genetics, growth, and development in children from birth through 21 years, and linking environmental exposures such as stress to phenotype and the presence of health disparities. The data can serve as a resource for highlighting diversity among people and their health outcomes, thus increasing the protection of public health in the United States. Last, the data can be a resource for health disparities researchers who prioritize the consideration of environmental and social factors when examining the influence of behavioral, biological and genetic differences on child health disparities.

## Author Contributions

SH drafted the initial text based on discussions among all the authors. CH provided background, edits, references, and perspective. NS provided background, edits, references, and perspective. All authors contributed to the article and approved the submitted version.

## Conflict of Interest

The authors declare that the research was conducted in the absence of any commercial or financial relationships that could be construed as a potential conflict of interest.

## Publisher's Note

All claims expressed in this article are solely those of the authors and do not necessarily represent those of their affiliated organizations, or those of the publisher, the editors and the reviewers. Any product that may be evaluated in this article, or claim that may be made by its manufacturer, is not guaranteed or endorsed by the publisher.
